# Supplementary figures and images for: Margelopsid species search taxonomic home within Corymorphidae and Boreohydridae
Source: PeerJ. 2023 Dec 4;11:e16265. doi: 10.7717/peerj.16265 (PMC10702351; doi:10.7717/peerj.16265)

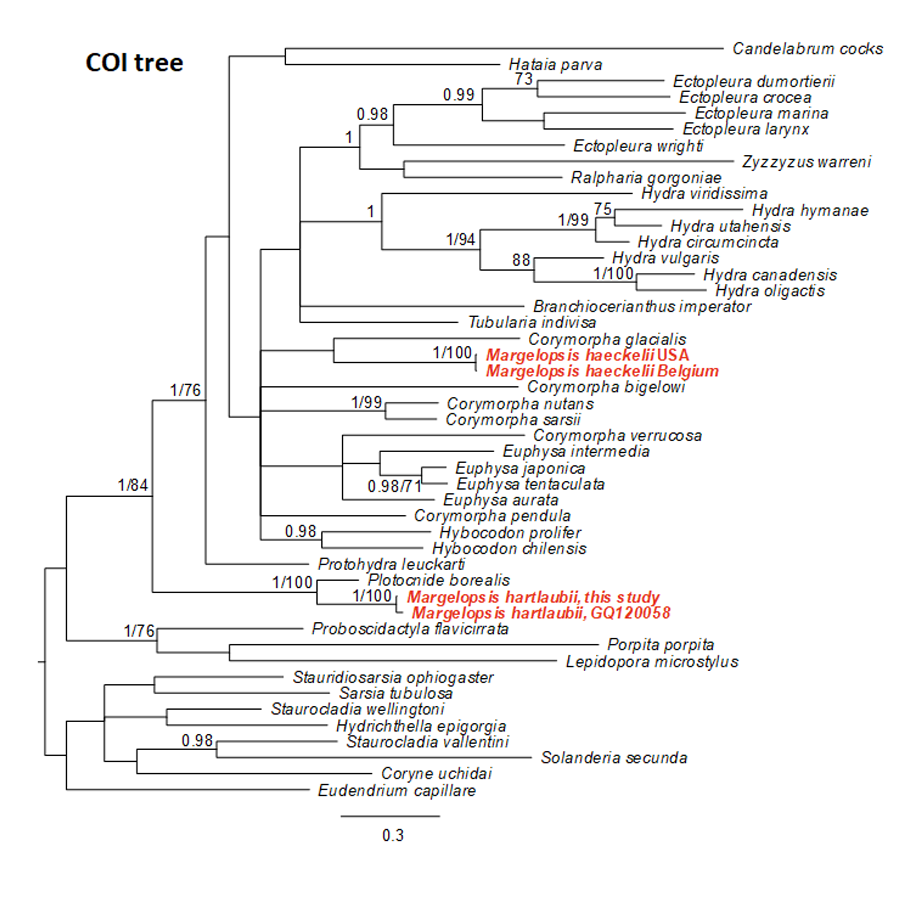

Supplement: Figure S1 — Node values indicate posterior probabilities (p > 0.95) and bootstrap values (ML¿70). Margelopsis haeckelii and Margelopsis hartlaubii are in red. [file peerj-11-16265-s001.png]

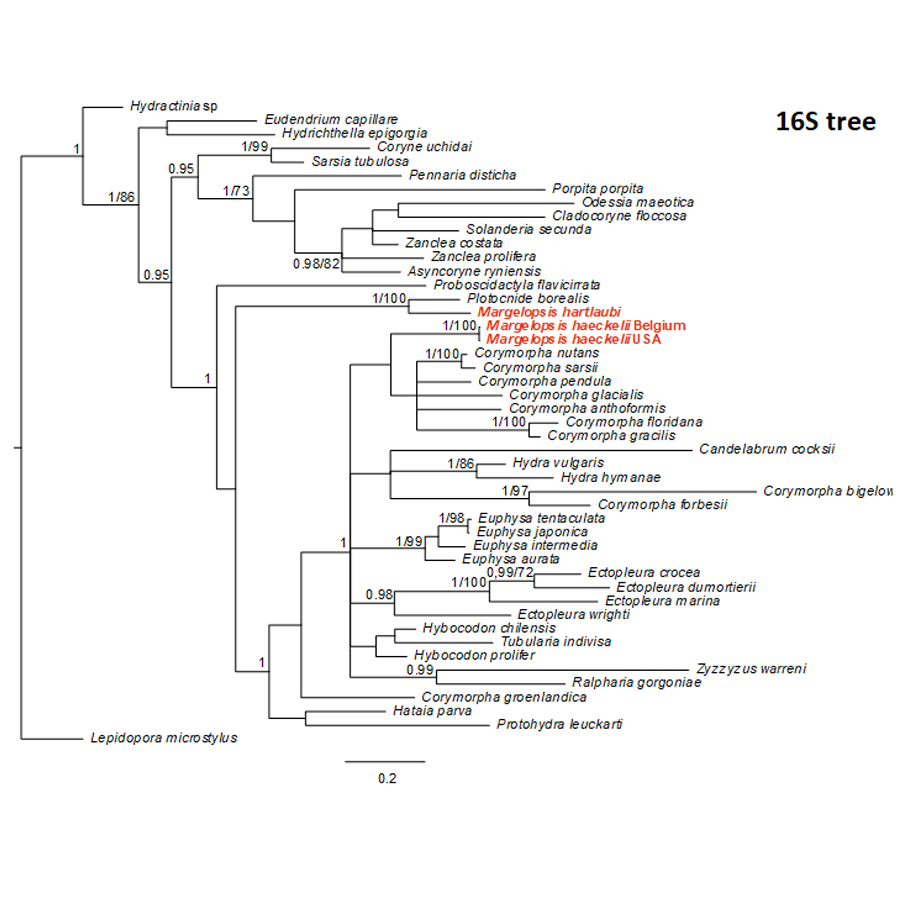

Supplement: Figure S2 — Node values indicate posterior probabilities (p > 0.95) and bootstrap values (ML¿70). Margelopsis haeckelii and Margelopsis hartlaubii are in red. [file peerj-11-16265-s002.png]

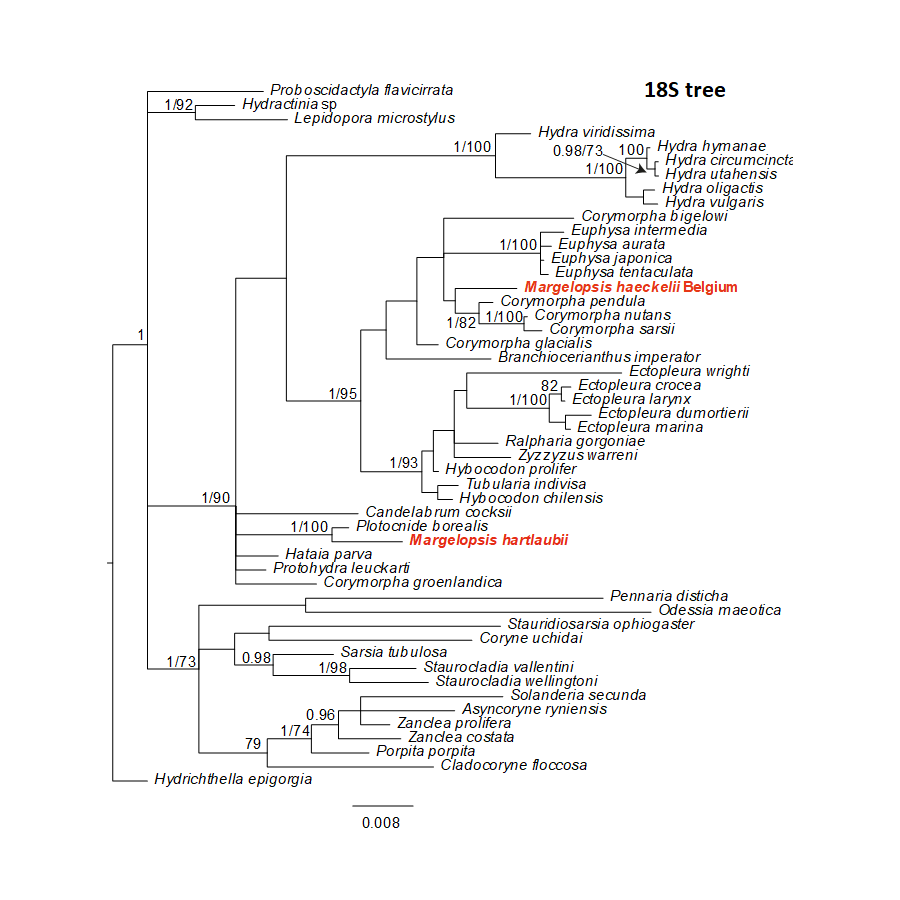

Supplement: Figure S3 — Node values indicate posterior probabilities (p > 0.95) and bootstrap values (ML >70). Margelopsis haeckelii and Margelopsis hartlaubii are in red. [file peerj-11-16265-s003.png]

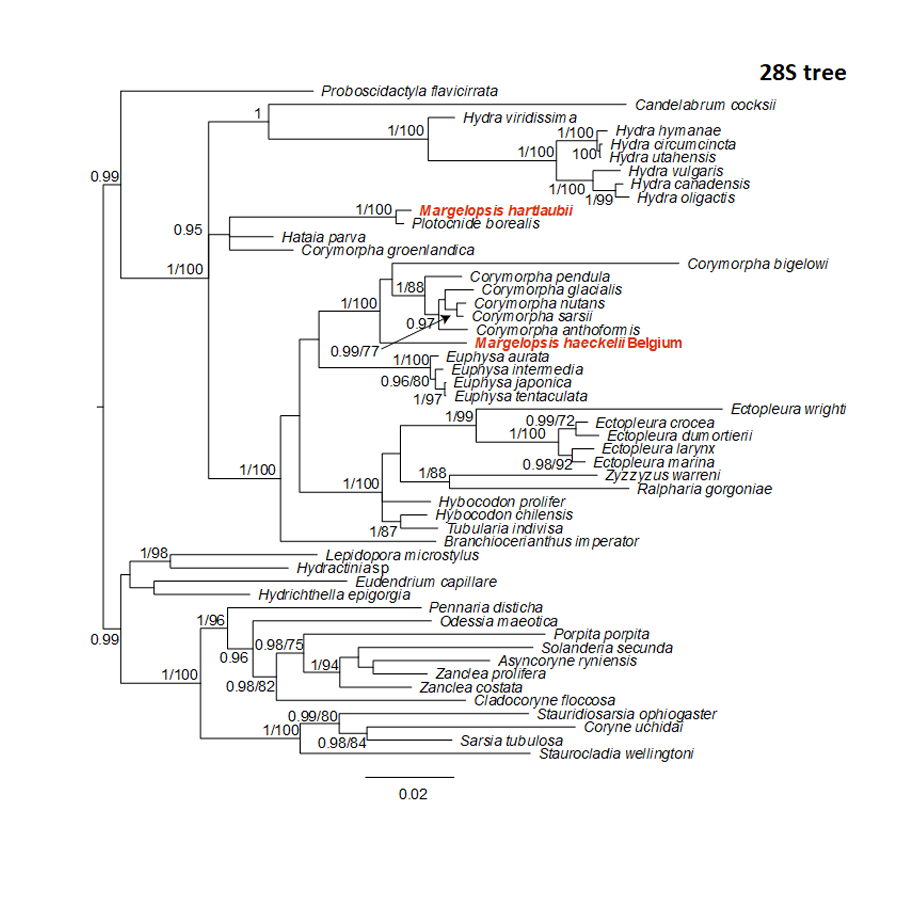

Supplement: Figure S4 — Node values indicate posterior probabilities (p > 0.95) and bootstrap values (ML >70). Margelopsis haeckelii and Margelopsis hartlaubii are in red. [file peerj-11-16265-s004.png]
