# Supplementary material for: Margelopsid species search taxonomic home within Corymorphidae and Boreohydridae
Source: PeerJ. 2023 Dec 4;11:e16265. doi: 10.7717/peerj.16265 (PMC10702351; doi:10.7717/peerj.16265)
Supplement: Table S1 — *We omitted species Euphysa monotentaculata Zamponi, 1983 in table because description is not available:This species is too poorly described to be identified, according to Brinckmann-Voss & Arai, 1998, and may be synonym to Euphysa aurata, according to Oliveira et al., 2016. [file peerj-11-16265-s005.docx]

| Species  according to Worms | Alternative species name | Hydroid morphology  if known | | | | Gonophore type | References |
| --- | --- | --- | --- | --- | --- | --- | --- |
|  |  | Size | Hydranth characters: diaphragm and tentacles | Hydrocaulus and gastral canals in hydrocaulus | Adhesive appendages of hydrocaulus |  |  |
| *Corymorpha abaxialis* (Kramp, 1962) | *Euphysora abaxialis* | hydroid unknown |  |  |  | medusa | Kramp, 1962; Kramp, 1968 |
| *Corymorpha adventitia* Fraser, 1941 | - | up to 20 mm | ? | with gastral canals in hydrocaulus (see Fraser, 1941 Plate 13 fig. 3) | with adventitious shoots from the main hydrocaulus | cryptomedusoid | Fraser, 1941; Vervoort, 2009 |
| *Corymorpha annulata* (Kramp, 1928) | *Euphysora annulata* | hydroid unknown | ? | ? | ? | medusa | Kramp, 1928; Kramp, 1968; Schuchert, 2010 |
| *Corymorpha anthoformis* (Yamada, 1974) | *Fukaurahydra* *anthoformis* | up to 20 mm | without diaphragm, gastral cavity sends in direction of aboral tentacles eight dichotomously branched radial canals | short hydrocaulus with parenchymous  endoderm, without having any distinct longitudinal canals | with many root-like processes are projected  from the base of the hydrocaulus | sporosac | Yamada et al., 1977 |
| *Corymorpha apiciloculifera* (Xu & Huang, 2003) | *Euphysora apiciloculifera* | hydroid unknown | ? | ? | ? | medusa | Xu and Huang, 2003; Du et al., 2012 |
| *Corymorpha balssi* Stechow, 1932 | may be *Zyzzyzus* sp. | infertile  colony | ? | hydrocaulus with longitudinal canals | with basal processes | unknown | Stechow, 1932; Ruthensteiner et al., 2008;  Watson et al., 2008;  Brinckmann-Voss, Calder, 2013 |
| *Corymorpha bigelowi* (Maas, 1905) | *Euphysora bigelowi* | up to 13 mm | with diaphragm, oral tentacles may be somewhat thickened at their tips | endodermal canals are visible in the hydrocaulus | anchoring  rootlets arise from the canals | medusa | Maas, 1905; Kramp, 1965, 1968; Sassaman, Rees, 1978; Schuchert, 2010 |
| *Corymorpha bitungensis* (Xu, Huang & Guo, 2013) | *Euphysora bitungensis* | hydroid unknown | ? | ? | ? | medusa | Lin et al., 2013 |
| *Corymorpha brunnescentis* (Huang, 1999) | *Euphysora brunnescentis* | hydroid unknown | ? | ? | ? | medusa | Huang, 1999 |
| *Corymorpha cargoi* (Vargas-Hernandez and Ochoa-Figueroa, 1991) | - | hydroid unknown | ? | ? | ? | medusa | Vargas-Hernandez and Ochoa-Figueroa, 1991; López-Pérez et al., 2022 |
| *Corymorpha carnea* (Clark, 1877) | *Rhizonema carnea* | up to 150 mm | ? | hydrocaulus with many partly anastomosing canals | with rooting filaments at the middle of basal spindle-shaped part of hydrocaulus | eumedusoid | Clark, 1877;  Hirohito, 1988;  Vervoort, 2009 |
| *Corymorpha crassocanalis* (Xu & Huang, 2003) | *Euphysora crassocanalis* | hydroid unknown | ? | ? | ? | medusa | Xu and Huang, 2003 |
| *Corymorpha floridana* Schuchert & Collins, 2021 | - | hydroid unknown | ? | ? | ? | medusa | Schuchert, Collins, 2021 |
| *Corymorpha forbesii* (Mayer, 1894) | *Vannuccia forbesii* | up to 20-30 mm | with moniliform oral and filiform aboral tentacles | hydrocaulus with 3-4 gastrodermal longitudinal canals | at basal end rows of papillae budding along gastrodermal canals, growing  into numerous, long rooting filaments | medusa | Mayer, 1894; Schuchert, 2010; Schuchert, Collins, 2021 |
| *Corymorpha fujianensis* (Xu and Huang, 2006) | *Euphysora fujianensis* | hydroid unknown | ? | ? | ? | medusa | Xu and Huang, 2006; Du et al., 2012 |
| *Corymorpha furcata* (Kramp, 1948) | *Euphysora furcata* | hydroid unknown | ? | ? | ? | medusa | Kramp, 1948; Kramp, 1968 |
| *Corymorpha gemmifera* (Bouillon, 1978) | *Euphysora gemmifera* | hydroid unknown | ? | ? | ? | medusa | Bouillon, 1978; Kavvamura, Kubota, 2005 |
| *Corymorpha gigantea* (Kramp, 1957) | *Euphysora gigantea* | hydroid unknown | ? | ? | ? | medusa | Kramp, 1957; Kramp, 1968 |
| *Corymorpha glacialis* M. Sars, 1860 | *Monocaulus glacialis* | up to 140 mm | outline of the hydranth body potentially may be kidney-shaped in deep water specimens | hydrocaulus with about 20-24 gastral canals | with tiny papillary projections and adhesive filaments in the basal part | sporosac | Sars, 1860; Svoboda, Stepanjants, 2001; Vervoort, 2009; Schuchert, 2010 |
| *Corymorpha gracilis* (Brooks, 1883) | *Steenstrupia gracilis, Euphysora gracilis* | hydroid unknown | ? | ? | ? | medusa | Brooks, 1883; Schuchert, Collins, 2021 |
| *Corymorpha groenlandica* (Allman, 1876) | *Monocaulus groenlandica* | up to 100 mm | ? | hydrocaulus with 14-18 longitudinal canals | Basal end with numerous tangled, thin attachment filaments | sporosac | Allman GJ. 1876; Schuchert, 2010; Svoboda, Stepanjants, 2001;  not *Corymorpha* according Nawrockii et al., 2013 |
| *Corymorpha interogona* (Xu & Huang, 2003) | *Euphysora interogona* | hydroid unknown | ? | ? | ? | medusa | Xu and Huang, 2003; Du et al., 2012 |
| *Corymorpha januarii* Steenstrup, 1855 | - | up to 22 mm | ? | hydrocaulus with branching and anastomosing longitudinal  endodermal canals | with rooting filaments and papillae in basal part | free-swimming eumedusoid | Steenstrup, 1855; Da Silveira and Migotto, 1992; Vervoort, 2009; Genzano et al., 2009 |
| *Corymorpha juliephillipsi* (Gershwin, Zeidler & Davie, 2010) | *Euphysora juliephillipsi* | hydroid unknown | ? | ? | ? | medusa | Gershwin, Zeidler and Davie, 2010 |
| *Corymorpha knides* (Huang, 1999) | *Euphysora knides* | hydroid unknown | ? | ? | ? | medusa | Huang , 1999;  Wang et al., 2019; |
| *Corymorpha luoyuanensis* Xu, Huang & Yang, 2022 | *Euphysora luoyuanensis* | hydroid unknown | ? | ? | ? | medusa | Liu et al., 2022 |
| *Corymorpha macrobulbus* (Xu & Huang, 2003) | *Euphysora macrobulbus* | hydroid unknown | ? | ? | ? | medusa | Xu and Huang, 2003; Du et al., 2012; Xu et al., 2014: fig. 243 |
| *Corymorpha meijiensis* (Xu, Huang & Guo, 2013) | *Euphysora meijiensis* | hydroid unknown | ? | ? | ? | medusa | Du et al, 2013 |
| *Corymorpha microrhiza* (Hickson & Gravely, 1907) | *Lampra microrhiza* | up to 120 mm | with filiform oral and aboral tentacles, with diaphragma | hydrocaulus with gastral canals | with delicate attachment processes (rooting filaments) at the proximal end | cryptomedusoid | Hickson and Gravely, 1907; Vervoort, 2009; Svoboda, Stepanjants, 2001 |
| *Corymorpha multiknoba* (Xu, Huang & Guo, 2014) | *Euphysora multiknoba* | hydroid unknown | ? | ? | ? | medusa | Xu et al., 2014: fig. 244 |
| *Corymorpha nana* Alder, 1857 | - | up to 18 mm | ? | up to 20 canals in hydrocaulus | with basal adhesive papillae | medusa | Alder, 1857;  Vervoort, 2009; Schuchert, 2010 |
| *Corymorpha nanhainesis* (Huang, Xu & Ling, 2012) | *Costa nanhainensis*. | hydroid unknown | ? | ? | ? | medusa | Huang et al, 2012 |
| *Corymorpha normani* (Browne, 1916) | *Steenstrupia normani* | hydroid unknown | ? | ? | ? | medusa | Browne, 1916; Kramp, 1968 |
| *Corymorpha nutans* M. Sars, 1835 | *Steenstrupia nutans* | up to 120 mm | ? | hydrocaulus with parenchymatic tissue and longitudinal canals | Basal part of hydrocaulus with tiny papillary projections more basally developing into long, fine rooting filaments | medusa | Sars, 1835; Kramp, 1968;  Vervoort, 2009; Schuchert, 2010 |
| *Corymorpha palma* Torrey, 1902 |  | up to 100 mm | ? | hydrocaulus with caenosarcal canals | filamentous rootlets arising on the proximal caenosarcal canals. The proximal ones are longest | attached eumedusoid | Torrey, 1902; Vervoort, 2009 |
| *Corymorpha pendula* L. Agassiz, 1862 | *Hybocodon pendulus, Hybocodon pendula* | up to 100 mm | ? | hydrocaulus with longitudinal canals | hydrocaulus is anchored by a number of root-like, tubular, fleshy processes (elongate filaments) | medusa | Agassiz, 1862: p.276; Kramp, 1961: p. 42-43; Mayer, 1910: p. 41-42 |
| *Corymorpha pileiformis* (Xu, Huang & Guo, 2014) | *Euphysora pileiformis* | hydroid unknown | ? | ? | ? | medusa | Xu et al., 2014 fig.245 |
| *Corymorpha pseudoabaxialis* (Bouillon, 1978) | *Euphysora pseudoabaxialis* | hydroid unknown | ? | ? | ? | medusa | Bouillon, 1978 |
| *Corymorpha rubicincta* Watson, 2008 |  | up to 40 mm | ? | hydrocaulus cylindrical, with a transverse red band (‘primary band’) at  one third of its height | base of hydrocaulus with numerous  papillae, attached in sediment by means of  thin rooting filaments. | cryptomedusoid | Watson, 2008; Vervoort, 2009 |
| *Corymorpha russelli* (Hamond, 1974) | *Euphysora russelli* | hydroid unknown | ? | ? | ? | medusa | Hamond, 1974 |
| *Corymorpha sagamina* Hirohito, 1988 |  | up to 40 mm | ? | Distal one third of hydrocaulus transparent with about 10 longitudinal canaliculi, remaining part of hydrocaulus not transparent with ten longitudinal ridges | Two longitudinal rows of alternate papillae present on each ridge, most developed at the middle portion. Below middle portion papillae become long anchoring filaments | free medusa with unknown morphology | Hirohito, 1988; Vervoort, 2009 |
| *Corymorpha sarsii* Steenstrup, 1855 | *Lampra sarsii*, *Monocaulus sarsii* | up to 100 mm | trophosome indistinguishable from *C. nutans* | hydrocaulus with about 10 longitudinal canals | Basal part of hydrocaulus with tiny papillary projections more basally developing into long, fine rooting filaments | medusoids | Vervoort, 2009; Schuchert, 2010 |
| *Corymorpha similis* (Kramp, 1959) | *Gotoea similis* | hydroid unknown | ? | ? | ? | medusa | Kramp, 1959: p.90, pl. II fig. 1 |
| *Corymorpha solidonema* (Huang, 1999) | *Euphysora solidonema* | hydroid unknown | ? | ? | ? | medusa | Huang, 1999; Xu et al., 2014: fig.246 |
| *Corymorpha symmetrica* Hargitt, 1924 | - | up to 30 mm | ? | hydrocaulus with caenosarcal canals | with stoloniferous growths from its base forming apparently a distinct rhizocaulus | medusoid? no specimen shows the character of manubrium or sex | Hargitt, 1924 p. 473-474 |
| *Corymorpha taiwanensis* (Xu & Huang, 2003) | *Euphysora taiwanensis* | hydroid unknown | ? | ? | ? | medusa | Xu and Huang, 2003; Du et al., 2012 |
| *Corymorpha tomoensis* Ikeda, 1910 | - | up to 34 mm | the septal structures separating the two hydranth-cavities as also that which separates the lower hydranth-cavity from the hydrocaulus-cavity | hydrocaulus with about 10 canals | bulbous end of hydrocaulus invested  with filaments (elongated papillae) | Gonophore may develop into free medusae | Ikeda, 1910;  Vervoort, 2009 |
| *Corymorpha typica* (Uchida, 1927) | *Gotoea typica* | hydroid unknown | ? | ? | ? | medusa | Uchida, 1927; Kramp, 1959, 1965 |
| *Corymorpha uvularis* (Fraser, 1941) | *Lampra uvularis* | up to 22 mm | ? | without evident canals in hydrocaulus (see Fraser, 1941 Plate 14 fig. 4) | ? | reduced gonophores shows no sign of tentacular processes | Vervoort, 2009; Fraser, 1941 |
| *Corymorpha vacuola* (Xu, Huang & Guo, 2012) | *Euphysora vacuola* | hydroid unknown | ? | ? | ? | medusa | Du et al, 2012 |
| *Corymorpha valdiviae* (Vanhoffen, 1911) | *Euphysora valdiviae* | hydroid unknown | ? | ? | ? | medusa | Vanhoffen, 1911; Kramp, 1968 |
| *Corymorpha verrucosa* (Bouillon, 1978) | *Euphysora verrucosa* | hydroid unknown | ? | ? | ? | medusa | Bouillon, 1978; Wang et al., 2019 |
| *Euphysa aurata* Forbes, 1848 | *-* | Up to 4,5 mm | hydranth without gastric diaphragm.  Oral capitate tentacles, moniliform aboral tentacles | hydrocaulus without longitudinal canals | at the aboral end of hydranth one or two whorls of  3-6 papillae, sometimes also a few similar papillae on stem,  in lower part up to 10 scattered attachment filaments, basal end may terminate in stolon-like  processes | medusa | Schuchert, 2010 |
| *Euphysa brevia* (Uchida, 1947) | *Sarsia brevia* | hydroid unknown | ? | ? | ? | medusa | Uchida, 1947 |
| *Euphysa flammea* (Hartlaub, 1902) | *-* | hydroid unknown | ? | ? | ? | medusa | Schuchert, 2010 |
| *Euphysa intermedia* (Schuchert, 1996) | *Corymorpha intermedia* | hydroid unknown | ? | ? | ? | medusa | Schuchert, 1996 |
| *Euphysa japonica* (Maas, 1909) | *Sarsia japonica* | hydroid unknown | ? | ? | ? | medusa | Maas, 1909 |
| *Euphysa peregrina* (Murbach, 1899) | *Hypolytus peregrinus* | up to 15 mm  temporarily  attached to substrata, may be found floating at the surface of the water | oral and aboral tentacles of the same shape and structure are slightly enlarged at the end | ? | A very delicate perisarcal envelope covers the whole hydrocaulus,  adhering to foreign objects. Where the hydranth joins the hydrocaulus there is a ring-like expansion | reduced gonophore | Murbach, 1899 |
| *Euphysa problematica* Schuchert, 1996 | *-* | hydroid unknown | ? | ? | ? | medusa | Schuchert, 1996 |
| *Euphysa ruthae* Norenburg & Morse, 1983 | *-* | up to 40 mm | oral tentacles capitate and moniliform with two annuli of nematocysts, aboral tentacles moniliform with about 15-25 annuli and a terminal knob | hydrocaulus without longitudinal canals | Just below the constriction,  the hydrocaulus bears 5-14 irregularly distributed papillae. May be numerous filaments proximal to the papillae. Hydrocaulus is loosely invested with a mucoid sheath. | sexual individuals unknown | Norenburg and Morse, 1983 |
| *Euphysa scintillans* Gershwin, Zeidler & Davie, 2010 | *-* | hydroid unknown | ? | ? | ? | medusa | Gershwin et al., 2010 |
| *Euphysa tentaculata* Linko, 1905 | *-* | hydroid unknown | ? | ? | ? | medusa | Schuchert, 2010 |
| *Euphysa tetrabrachia* Bigelow, 1904 | *-* | hydroid unknown | ? | ? | ? | medusa | Bigelow, 1904 |
| *Euphysa vervoorti* Brinckmann-Voss & Arai, 1998 | *-* | hydroid unknown | ? | ? | ? | medusa | Brinckmann-Voss & Arai, 1998 |
| *Margelopsis haeckelii* Hartlaub, 1897 | *-* | up to 2 mm, planktonic polyp | two circlets of tentacles, All tentacles are more or less capitate, but have ring-shaped or spiral batteries of cnidosysts throughout their whole length (thus unclear moniliform?) | without longitudinal canals | the aboral  pole terminates in a short stalk with a sucker-shaped depression, without adhesive appendages | medusa | Werner, 1955; Schuchert, 2006;  Our data |

References:

Agassiz L. 1862. Contributions to the natural history of the United States of America. Little Brown, Boston. 4:1–380. available online at https://www.biodiversitylibrary.org/page/16068829

Alder J. 1857. VII. A Catalogue of the Zoophytes of Northumberland and Durham. By Joshua Alder, Esq. Read, at the Anniversary Meeting of the Club, May 15, 1856. From the “Transactions of the Tunesyde naturalists’ field club”. Newcastle-upon-Tyne: F.&W. Dodsworth, Collingwood street. 1–72 (93–154). available online at https://archive.org/details/b30473858/mode/1up

Allman GJ. 1876. Diagnoses of new genera and species of Hydroida. Journal of the Linnean Society of London 12:251–284. available online at https://www.biodiversitylibrary.org/page/31981814

Bigelow HB. 1904. Medusae from the Maldive Islands. Bulletin of the Museum of Comparative Zoology at Harvard College 39:245–269. available online at https://www.biodiversitylibrary.org/page/4199303

Bouillon J. 1978. Hydromeduses de la mer de Bismarck (Papouasie, Nouvelle-Guinée). Partie 1: Anthomedusae Capitata (Hydrozoa - Cnidaria). Cahiers de Biologie Marine 19:249–297.

Brinckmann-Voss A, and Arai MN. 1998. Further notes on Leptolida (Hydrozoa: Cnidaria) from Canadian Pacific waters. Zoologische Verhandelingen 323(5):37–68.

Brinckmann-Voss A, and Calder DR. 2013. Zyzzyzus rubusidaeus (Cnidaria, Hydrozoa, Tubulariidae), a new species of anthoathecate hydroid from the coast of British Columbia, Canada. Zootaxa 3666(3):389–397.

Brooks WK. 1883. List of medusae found at Beaufort, N.C., during the summers of 1880 and 1881. Studies from the Biological Laboratory, Johns Hopkins University 2:135–146. available online at http://www.archive.org/stream/studiesfrombiol00martgoog#page/n152/mode/1up

Browne ET. 1916. Medusae from the Indian Ocean (collected by Prof. Stanley Gardiner in H.M.S. Sealark in 1905). In: The Percy Sladen Trust Expedition to the Indian Ocean. In: Transactions of the Linnean Society of London, Zoology 17: 169–209. available online at https://www.biodiversitylibrary.org/page/25256788

Clark SF. 1877. Report on the hydroids collected on the coast of Alaska and the Aleutian Islands by W.H. Dall, U.S. Coast Survey, and party, from 1871 to 1874 inclusive. Proceedings of the Academy of Natural Sciences of Philadelphia 28:209–235. available online at https://biodiversitylibrary.org/page/26298960

Du F-Y, Xu Z-Z, Huang J-Q, Guo D-H. 2012. Studies on the medusae (Cnidaria) from the Beibu Gulf in the northern South China Sea, with description of three new species. Acta Zootaxonomica Sinica 37:506–519. available online at http://eng.med.wanfangdata.com.cn/PaperDetail.aspx?qkid=dwfl&qcode=dwfl201203006

Du F-Y, Lin Z-J, Xu Z-Z, Huang J-Q, Guo D-H. 2013. Three new species of hydroidomedusae (Cnidaria) from the Meiji Reef and Daya bay, South China Sea. Acta Zootaxonomica Sinica 38(4):749–755.

Fraser CM. 1941. New species of hydroids, mostly from the Atlantic Ocean, in the United States National Museum. Proceedings of the United States National Museum 91:77–89. available online at http://www.biodiversitylibrary.org/item/32533#103

Genzano G, Rodriguez C, Pastorino G, Mianzan H. 2009. The hydroid and medusa of Corymorpha januarii (Cnidaria: Hydrozoa) in temperate waters of the Southwestern Atlantic Ocean. Bulletin of Marine Science 84(2):229–235.

Gershwin L-A, Zeidler W, Davie PJF. 2010. Medusae (Cnidaria) of Moreton Bay, Queensland, Australia. Memoirs of the Queensland Museum 54: 47–108.

Hamond R. 1974. Some medusae and other Hydrozoa from the Indian Ocean and the Bass Strait. J. nat. Hist. 8:549–561.

Hargitt CW. 1924. Hydroids of the Philippine Islands. Philippine Journal of Science 24:467–507.

Hickson SJ, and Gravely FH. 1907. II Hydroid Zoophytes. National Antarctic Expedition 3: 1–33. available online at https://www.biodiversitylibrary.org/page/854217

Hirohito Emperor of Japan. 1988. The hydroids of Sagami Bay. (Part 1. Athecata). Publs Biol. Lab., Imp. Household, Tokyo 1–179.

Huang J. 1999. Three new species of genus Euphysora from China seas (Hydrozoa: Anthomedusae, Corymorphidae). Acta Oceanol. Sinica (English Edit.) 18(3):435–441.

Huang J-Q, Xu Z-Z, Lin M, Guo D-H, Wang Ch-G, Xue W-L. 2012. One new genus, two new species and one new record of Corymorphidae from the South China Sea (Anthomedusae, Capitata). Acta Zootaxonomica Sinica 37:520–524.

Ikeda J. 1909. On a new species of Corymorpha from Japan. Annotnes zool. japon. 7(3):153–164.

Kavvamura M, and Kubota S. 2005. First occurrence of Euphysora gemmifera (Cnidaria, Hydrozoa, Corymorphidae) in Japan. Biogeography 7:31–33.

Kramp PL. 1928. Papers from Dr. Mortensen's Pacific Expeditions 1914-1916, XLIII. Hydromedusae 1. Anthomedusae. Vidensk. Meddr dansk naturh. Foren. 85:27–64.

Kramp PL. 1948. Trachymedusae and Narcomedusae from the "Michael Sars" North Atlantic deep-sea Expedition 1910, with additions on Anthomedusae, Leptomedusae, and Scyphomedusae. Report on the scientific results of the "Michael Sars" north Atlantic deep-sea expedition 1910. 5(9):1–23.

Kramp PL. 1957. Hydromedusae from the Discovery collections. Discovery Rep. 29:1–128. available online at http://www.biodiversitylibrary.org/page/5568717#page/15/mode/1up

Kramp PL. 1959. The Hydromedusae of the Atlantic Ocean and adjacent waters. Dana Report 46:1–283.

Kramp PL. 1961. Synopsis of the medusae of the world. Journal of the marine biological Association of the United Kingdom 40:7–382.

Kramp PL. 1962. Medusae of Vietnam. Videnskabelige Meddelelser fra Dansk naturhistorisk Forening i København 124:305–366.

Kramp PL. 1965. The hydromedusae of the Pacific and Indian Oceans. Dana Report 68:1–162.

Kramp PL. 1968. The hydromedusae of the Pacific and Indian Oceans. Dana Report 72:1–200.

Lin M., Xu Z.-Z., Huang J.-Q., Nurul F., Guo D.-H., Wang C.-G., Chen B. (2013). Two new species of Anthomedusae from the Bitung Strait, Indonesia (Cnidaria). Acta Zootaxonomica Sinica. 38(2): 246–250.

Liu ZY, Yang YY, Xu ZZ, Huang JQ. 2022. A new species of medusae from Luoyuan Bay, Fujian, China. Zoological Systematics 47(4):345–348.

López-Pérez A, Reyes-Bonilla H, Calderón-Aguilera LE, Cruz-Piñón G, Cupul-Magaña A, Medina-Rosas P, Mora-Vallín Z, Meléndez-Rosas R. 2022. Cnidarios (Cnidaria). In Bastida-Zavala JR, & del Socorro García-Madrigal M (Eds.) “Invertebrados marinos y costeros del Pacífico sur de México”, Universidad del Mar Puerto Ángel, Oaxaca, pp. 31–53.

Maas O. 1905. Die Craspedoten Medusen der Siboga-Expeditie. Siboga Expeditie 10:1–84. available online at https://www.biodiversitylibrary.org/page/11712344

Maas O. 1909. Japanische medusen. Series: Beiträge zur naturgeschichte Ostasiens. Abhandlungen der bayerischen Akademie der Wissenschaften, mathematisch physikalische Classe, Supplement Band 1:1–53. available online at https://www.biodiversitylibrary.org/page/5542853

Mayer AG. 1894. An account of some medusae obtained in the Bahamas. In: Cruise of the Steam Yacht "Wild-Duck" in the Bahamas, January to April 1893, in charge of Alexander Agassiz. Bulletin of the Museum of comparative Zoölogy of Harvard College 25(11):235–242. available online at http://www.biodiversitylibrary.org/item/24909

Mayer AG. 1910. Medusae of the World. Published by the Carnegie Institution of Washington. Publication No 109, Vol. 1, available online at https://doi.org/10.5962/bhl.title.5996

Murbach L. 1899. Hydroids from Wood's Hole. Hypolytus peregrinus, a new unattached marine hydroid: Corinitis Agassizii and its medusa. Q. Jl microsc. Sci., n. ser. 42. 3:341–360.

Nawrocki AM, Collins AG, Hirano YM, Schuchert P, Cartwright P. 2013. Phylogenetic placement of Hydra and relationships within Aplanulata (Cnidaria: Hydrozoa). Molecular Phylogenetics and Evolution 67(1):60–71.

Norenburg, J.L. and Morse, M.P., 1983. Systematic implications of Euphysa ruthae n. sp.(Athecata: Corymorphidae), a psammophilic solitary hydroid with unusual morphogenesis. Transactions of the American Microscopical Society, pp.1-17.

Ruthensteiner B, Reinicke GB, & Straube N. 2008. The type material of Hydrozoa described by Eberhard Stechow in the Zoologische Staatssammlung München. Spixiana 31(1):3–27.

Sars M. 1835. Beskrivelser og Iagttagelser over nogle moerkelige eller nye i Havet ved den Bergenske Kyst levende Dyr af Polypernes, Acalephernes, Radiaternes, Annelidernes og Molluskernes classer, med en kort Oversigt over de hidtil af Forfatteren sammesteds fundne Arter og deres Forekommen. [book]. Thorstein Hallagers Forlag hos Chr. Dahl, R.S., xii + 81 pp., 15 plates, Bergen. available online at https://doi.org/10.5962/bhl.title.13017

Sars M. 1860. Udtog af en Afhandling, som med de tilhörende talrige Afbildninger er bestemt for naeste Hefte af Fauna littoralis Norvegiae, om Ammeslaegten Corymorpha og dens Arter samt de af disse opammede Meduser. Forhandlinger i Videnskapsselskapet i Kristiania 1859. 96–105. available online at https://www.biodiversitylibrary.org/page/22192331

Sassaman C, and Rees JT. 1978. The life cycle of Corymorpha (= Euphysora) bigelowi (Maas, 1905) and its significance in the systematics of corymorphid hydromedusae. The Biological Bulletin 154(3):485–496.

Schuchert P. 1996. The marine fauna of New Zealand: Athecate hydroids and their medusae (Cnidaria: Hydrozoa). New Zealand oceanographic institute memoir 106:1–159.

Schuchert P. 2006. The European athecate hydroids and their medusae (Hydrozoa, Cnidaria): Capitata part 1. Revue suisse de Zoologie 113(2):325–410. DOI: 10.5962/bhl.part.80356

Schuchert P. 2010. The European athecate hydroids and their medusae (Hydrozoa, Cnidaria): Capitata part 2. Revue suisse de Zoologie 117(3):337–555.

Schuchert P, Collins R. 2021. Hydromedusae observed during night dives in the Gulf Stream. Revue suisse de Zoologie 128(2):237–356. available online at https://doi.org/10.35929/rsz.0049

Silveira FD, Migotto AE. 1992. Rediscovery of Corymorpha januarii Steenstrup, 1854 (Hydrozoa, Corymorphidae) on the southeastern and southern coasts of Brazil. Steenstrupia 18(4):81–9.

Stechow E. 1932. Neue Hydroiden aus dem Mittelmeer und dem Pazifischen Ozean, nebst Bemerkungen über einige wenig bekannte Formen. Zoologischer Anzeiger 100:81–92.

Steenstrup JJS. 1855. En ny og tropisk Art af Smaagoplernes Ammeslaegt: Corymorpha Sars (Corym. Januarii Stp.). Vidensk. Meddr dansk naturh. Foren. 1–3:46–48.

Svoboda A,and Stepanjants SD. 2001. Redescription of two Antarctic Corymorphidae species and the reestablishment of the genus Monocaulus (Cnidaria: Hydrozoa). Marine Ecology 22(1‐2):53–70.

Torrey HB. 1902. The Hydroida of the Pacific Coast of North America. University of California Publications Zoology. 1: 1–104. available online at https://biodiversitylibrary.org/page/29942277

Uchida T. 1927. Studies on Japanese hydromedusae. I. Anthomedusae. Journal of the Faculty of Science, Imperial University of Tokyo, Section IV, Zoology. 1:145–241.

Uchida T. 1947. Some medusae from the Central Pacific. Journal of the Faculty of Science, Imperial University of Tokyo, Zoology 7:297–319.

Vanhöffen E. 1911. Die Anthomedusen und Leptomedusen der Deutschen Tiefsee Expedition 1898-1899. Wissenschaftliche Ergebnisse der deutschen Tiefsee Expedition Valdivia 19:193–233.

Vargas-Hernández JM, and E. Ochoa-Figueroa. 1991. Un nuevo género y descripción de una nueva especie para la familia Tubulariidae (Hidrozoa: Anthomedusae) en el Pacífico méxicano. A new genus and description of a new species for the family Tubulariidae (Hydrozoa, Anthomedusae) in the Mexican Pacific. Brenesia 33:75–80.

Vervoort W. 2009. Corymorpha tomoensis Ikeda, 1910 (Cnidaria, Hydrozoa): first record of a corymorphid hydropolyp from Indonesian waters and a review of the species of Corymorpha M. Sars, 1835. Zoologische Mededelingen 83(21):759–776.

Wang X, Lin K, Xu Z, Guo D, Huang J. 2019. Some new Hydroidomedusa (Cnidaria) from the northern South China Sea. Zoological Systematics 44(3):191–205. DOI: 10.11865/zs.201923

Watson JE. 2008. Corymorpha rubicincta, a new Hydroid (Hydrozoa, Anthoathecata, Corymorphidae) from Port Phillip, Australia. Memoirs of Museum Victoria 65:185–188.

Werner B. 1955. On the development and reproduction of the anthomedusan Margelopsis haeckeli Hartlaub. Annals of the New York Academy of Sciences 62(1):3–29. doi: 10.1111/j.1749-6632.1955.tb35352.x

Xu Z, Huang J. 2003. On new species and records of Euphysora in Taiwan Strait and its adjacent waters J. Oceanogr. Taiwan Strait = Taiwan Haixia 22(2):136–144.

Xu ZZ, Huang JQ. 2006. On new genus, species and record of Laingiomedusae and Anthomedusae in Fujian coast (Cnidaria, Hydroidomedusae). Journal of Xiamen University Natural Science 45:233–249.

Xu Z-Z, Huang J-Q, Lin M, Guo D-H, Wang C-G. 2014. The superclass Hydrozoa of the Phylum Cnidaria in China. China Ocean Press, Bejing. 1:1–456, 2:495–945.

Yamada M, Konno K, Kubota S. 1977. On a new athecate hydroid, Fukaurahydra anthoformis n. gen. n. sp., from northern Japan. Proc. jap. Acad. Sci. 53(3):151–154.
