# Supplementary material for: Margelopsid species search taxonomic home within Corymorphidae and Boreohydridae
Source: PeerJ. 2023 Dec 4;11:e16265. doi: 10.7717/peerj.16265 (PMC10702351; doi:10.7717/peerj.16265)
Supplement: Table S2 — *We omitted species Euphysa monotentaculata Zamponi, 1983 in table because description is not available:This species is too poorly described to be identified, according to Brinckmann-Voss & Arai, 1998, and may be synonym to Euphysa aurata, according to Oliveira et al., 2016. [file peerj-11-16265-s006.docx]

| Species  according to Worms | Alternative species name | medusa morphology | | | | References |
| --- | --- | --- | --- | --- | --- | --- |
|  |  | Principal Tentacle | Reduced tentacles | Apex of the bell | Apical canal |  |
| *Corymorpha abaxialis* (Kramp, 1962) | *Euphysora abaxialis* | moniliform with abaxial row of nematocyst knobs and with terminal nematocyst knob | tentacles rudimentary | rounded apex | absent | Kramp, 1962; Kramp, 1968 |
| *Corymorpha adventitia* Fraser, 1941 | - | - | - | - | - | Fraser, 1941; Vervoort, 2009 |
| *Corymorpha annulata* (Kramp, 1928) | *Euphysora annulata* | moniliform with numerous rings of nematocysts, with terminal nematocyst knob | 3 short cone-shaped tentacles | pointed apex | + | Kramp, 1928; Kramp, 1968; Schuchert, 2010 |
| *Corymorpha anthoformis* (Yamada, 1974) | *Fukaurahydra* *anthoformis* | - | - | - | - | Yamada et al., 1977 |
| *Corymorpha apiciloculifera* (Xu & Huang, 2003) | *Euphysora apiciloculifera* | principal tentacles with abaxial nematocyst knobs including semicyclic and normal nematocysts arrangement | one opposite  the main tentacle larger than the two others | with or without apical projection | with apical chamber | Xu and Huang, 2003; Du et al., 2012 |
| *Corymorpha balssi* Stechow, 1932 | may be *Zyzzyzus* sp. | - | - | - | - | Stechow, 1932; Ruthenstainer et al., 2008;  Watson et al., 2008;  Brinckmann-Voss, Calder, 2013 |
| *Corymorpha bigelowi* (Maas, 1905) | *Euphysora bigelowi* | moniliform with adaxial nematocyst clusters and terminal knob of nematocysts | 3 short pointed tentacles without nematocyst clusters | pointed apex with small papillae | Variable character: apical canal is present or absent | Maas, 1905; Kramp, 1965, 1968; Sassaman, Rees, 1978; Schuchert, 2010 |
| *Corymorpha bitungensis* (Xu, Huang & Guo, 2013) | *Euphysora bitungensis* | moniliform tentacle with terminal knob of nematocysts | 3 short filiform tentacles | with apical projection | with apical canal | Lin et al., 2013 |
| *Corymorpha brunnescentis* (Huang, 1999) | *Euphysora brunnescentis* | long tentacle with 50-60 abaxial nematocyst knobs and with bulb-like terminal knob | bulbs with sickle-shaped structure and with pigment spots | umbrella nearly spherical, without apical projection | without apical canal, with rounded apical chamber | Huang, 1999 |
| *Corymorpha cargoi* (Vargas-Hernandez and Ochoa-Figueroa, 1991) | - | with large terminal knob of nematocysts | absent | with pointed apical projection | without apical canal (?) | Vargas-Hernandez and Ochoa-Figueroa, 1991; López-Pérez et al., 2022 |
| *Corymorpha carnea* (Clark, 1877) | *Rhizonema carnea* | - | eumedusoid with three tentacular buds and a short, rudimentary  tentacle | - | - | Clark, 1877;  Vervoort, 2009 |
| *Corymorpha crassocanalis* (Xu & Huang, 2003) | *Euphysora crassocanalis* | the row of nematocyst knobs on principal tentacle adaxial or lateral | ? | ? | ? | Xu and Huang, 2003 |
| *Corymorpha floridana* Schuchert & Collins, 2021 | - | moniliform with terminal knob of nematocysts | filiform tentacle and two tentacle stumps | pointed apex | absent | Schuchert, Collins, 2021 |
| *Corymorpha forbesii* (Mayer, 1894) | *Vannuccia forbesii* | stiff tentacle with club-like terminal swelling | absent | without apical process | absent | Mayer, 1894; Schuchert, 2010; Schuchert, Collins, 2021 |
| *Corymorpha fujianensis* (Xu and Huang, 2006) | *Euphysora fujianensis* | principal tentacle moniliform with 4－5 ring nematocysts | other whole tentacles have over 16 spherical nematocyst knobs | ? | ? | Xu and Huang, 2006; Du et al., 2012 |
| *Corymorpha furcata* (Kramp, 1948) | *Euphysora furcata* | tentacle twice bifurcated with four terminal knobs of nematocysts. | filiform tentacle and two ones short and conical | pointed apex | stomach with broad conical apical chamber | Kramp, 1948; Kramp, 1968 |
| *Corymorpha gemmifera* (Bouillon, 1978) | *Euphysora gemmifera* | moniliform with about twenty clusters  abaxial nematocysts | absent | rounded apex | absent | Bouillon, 1978; Kavvamura, Kubota, 2005 |
| *Corymorpha gigantea* (Kramp, 1957) | *Euphysora gigantea* | tentacle with several bifurcated lateral branches | absent | globular bell | absent | Kramp, 1957; Kramp, 1968 |
| *Corymorpha glacialis* M. Sars, 1860 | *Monocaulus glacialis* | - | - | - | - | Sars, 1860; Svoboda, Stepanjants, 2001; Vervoort, 2009; Schuchert, 2010 |
| *Corymorpha gracilis* (Brooks, 1883) | *Steenstrupia gracilis, Euphysora gracilis* | moniliform with rings of nematocysts and with terminal nematocyst knob | filiform tentacle and two reduced cones | very  large, pointed apical process | apical canal entering apical process | Brooks, 1883; Schuchert, Collins, 2021 |
| *Corymorpha groenlandica* (Allman, 1876) | *Monocaulus groenlandica* | - | - | - | - | Allman, 1876; Schuchert, 2010; Svoboda, Stepanjants, 2001;  not *Corymorpha* according Nawrockii et al., 2013 |
| *Corymorpha interogona* (Xu & Huang, 2003) | *Euphysora interogona* | long moniliform tentacle with over 60 abaxial spherical nematocyst knobs | three very small marginal bulbs rudimentary | ? | ? | Xu and Huang, 2003; Du et al., 2012 |
| *Corymorpha januarii* Steenstrup, 1855 | - | - | - | rounded apical process | short conical apical canal | Steenstrup, 1855; Da Silveira and Migotto, 1992; Vervoort, 2009; Genzano et al., 2009 |
| *Corymorpha juliephillipsi* (Gershwin, Zeidler & Davie, 2010) | *Euphysora juliephillipsi* | moniliform with up to 12 rings and terminal knob of nematocysts | opposite filiform tentacle and two reduced stumps | with a very long, narrow  apical projection | with an off-centre long,  narrow apical canal | Gershwin, Zeidler and Davie, 2010 |
| *Corymorpha knides* (Huang, 1999) | *Euphysora knides* | very long, with 20–30 abaxial knobs of nematocysts and no clearly large terminal knob | absent | with a slight rounded apical  projection | with a distinct elliptical apical chamber (?) | Huang , 1999;  Wang et al., 2019; |
| *Corymorpha luoyuanensis* Xu, Huang & Yang, 2022 | *Euphysora luoyuanensis* | principal tentacle short and stiff, with 6–7 adaxial nematocysts  knobs and with a large terminal nematocyst knob | Three bulbs with a small  and short papilla-like tentacle | with a well developed blunt solid apical projection | without apical chamber | Liu et al., 2022 |
| *Corymorpha macrobulbus* (Xu & Huang, 2003) | *Euphysora macrobulbus* | moniliform with 3-6 small adaxial nematocyst knobs and with large terminal knob of nematocysts | 3 filiform tentacles, smallest – opposite one. | without apical projection | without apical canal | Xu and Huang, 2003; Du et al., 2012; Xu et al., 2014: fig. 243 |
| *Corymorpha meijiensis* (Xu, Huang & Guo, 2013) | *Euphysora meijiensis* | short and stiff with 4 adaxial nematocyst knobs and with a large terminal nematocyst knob | opposite filiform tentacle with large terminal red pigment patch and two reduced papilla-like tentacles | with cone-shaped solid apical projection | without apical chamber | Du et al, 2013 |
| *Corymorpha microrhiza* (Hickson & Gravely, 1907) | *Lampra microrhiza* | - | - | - | - | Hickson and Gravely, 1907; Vervoort, 2009; Svoboda, Stepanjants, 2001 |
| *Corymorpha multiknoba* (Xu, Huang & Guo, 2014) | *Euphysora multiknoba* | with 5-6 hemicyclic nematocyst clusters along abaxial surface of conical base and with over 100 abaxial nematocyst knobs along the tentacle, without large terminal knob of nematocysts | cone-shaped bulbs without tentacles | bell shaped with rounded top | absent | Xu et al., 2014: fig. 244 |
| *Corymorpha nana* Alder, 1857 | - | One tentacle | 3 tentacle rudiments | without apical process | without apical canal | Alder, 1857;  Vervoort, 2009; Schuchert, 2010 |
| *Corymorpha nanhainesis* (Huang, Xu & Ling, 2012) | *Costa nanhainensis*. | solid tentacle terminating in two large nematocyst knobs | 3 marginal bulbs without tentacles | Dome-shaped, without apical process | without apical canal | Huang et al, 2012 |
| *Corymorpha normani* (Browne, 1916) | *Steenstrupia normani* | with three lateral knobs of nematocysts, with large terminal nematocyst knob | absent | conical | broad apical chamber high and conical | Browne, 1916; Kramp, 1968 |
| *Corymorpha nutans* M. Sars, 1835 | *Steenstrupia nutans* | moniliform tentacle with 40-80 annular  nematocyst clusters, without terminal nematocyst knob | absent | pointed apical process | long apical canal | Sars, 1835; Kramp, 1968;  Vervoort, 2009; Schuchert, 2010 |
| *Corymorpha palma* Torrey, 1902 |  | - | - | - | - | Torrey, 1902; Vervoort, 2009 |
| *Corymorpha pendula* L. Agassiz, 1862 | *Hybocodon pendulus, H.pendula* | long moniliform tentacle | absent according to Mayer, 1910 | pyriform | without apical canal | Agassiz, 1862: p.276; Kramp, 1961: p. 42-43; Mayer, 1910: p. 41-42 |
| *Corymorpha pileiformis* (Xu, Huang & Guo, 2014) | *Euphysora pileiformis* | long tentacle, with over 20 abaxial knobs of nematocysts, without terminal knob of nematocysts | absent | with round apex | with ovaliform apical chamber, connected to upper part of manubrium by a short canal | Xu et al., 2014 fig.245 |
| *Corymorpha pseudoabaxialis* (Bouillon, 1978) | *Euphysora pseudoabaxialis* | Short tentacle with a dozen of spherical clusters abaxial nematocysts, without large terminal nematocyst knob | absent | rounded apex | absent | Bouillon, 1978 |
| *Corymorpha rubicincta* Watson, 2008 |  | - | - | - | - | Watson, 2008; Vervoort, 2009 |
| *Corymorpha russelli* (Hamond, 1974) | *Euphysora russelli* | moniliform tentacle with 9 globular clusters of nematocysts, the last one forming a terminal knob | two lateral filiform and reduced opposite tentacle | with rounded apical projection | without apical canal | Hamond, 1974 |
| *Corymorpha sagamina* Hirohito, 1988 |  | ? | ? | ? | ? | Hirohito, 1988; Vervoort, 2009 |
| *Corymorpha sarsii* Steenstrup, 1855 | *Lampra sarsii*, *Monocaulus sarsii* | - | - | - | - | Vervoort, 2009; Schuchert, 2010 |
| *Corymorpha similis* (Kramp, 1959) | *Gotoea similis* | long tentacle with large terminal knob of nematocysts | absent | pyriform, flat-topped | absent | Kramp, 1959: p.90, pl. II fig. 1 |
| *Corymorpha solidonema* (Huang, 1999) | *Euphysora solidonema* | short and stiff tentacle with over 10 ring of nematocysts and with large terminal nematocyst knob | two short pointed tentacles and smallest cone-shaped opposite tentacle | umbrella bell-shaped | without apical canal or apical chamber | Huang, 1999; Xu et al., 2014: fig.246 |
| corymorpha symmetrica Hargitt, 1924 | - | absent | absent | - | - | Hargitt, 1924 |
| *Corymorpha taiwanensis* (Xu & Huang, 2003) | *Euphysora taiwanensis* | moniliform tentacle with over 16 of spherical nematocyst knobs | absent | ? | ? | Xu and Huang, 2003; Du et al., 2012 |
| *Corymorpha tomoensis* Ikeda, 1910 |  | ? | ? | ? | ? | Ikeda, 1910; Vervoort, 2009 |
| *Corymorpha typica* (Uchida, 1927) | *Gotoea typica* | stiff tentacle with round terminal nematocyst knob | absent | pyriform, flat-topped | absent | Uchida, 1927; Kramp, 1959, 1965 |
| *Corymorpha uvularis* (Fraser, 1941) | *Lampra uvularis* | - | - | - | - | Vervoort, 2009; Fraser, 1941 |
| *Corymorpha vacuola* (Xu, Huang & Guo, 2012) | *Euphysora vacuola* | with 30－40 abaxial nematocyst knobs and without large terminal knob | absent | with a well developed rounded solid  apical projection | absent | Du et al, 2012 |
| *Corymorpha valdiviae* (Vanhoffen, 1911) | *Euphysora valdiviae* | short, twice bifurcated without nematocyst clusters | 3 short conical tentacles | short conical apex | large conical apical chamber | Vanhoffen, 1911; Kramp, 1968 |
| *Corymorpha verrucosa* (Bouillon, 1978) | *Euphysora verrucosa* | elongated tentacle, armed with about 30 abaxial capitations  and a terminal nematocyst knob | absent | rounded apex | absent | Bouillon, 1978; Wang et al., 2019 |
| *Euphysa aurata* Forbes, 1848 |  | With one moniliform  tentacle only | three non-tentacular bulbs | evenly rounded umbrella | without apical canal | Schuchert, 2010 |
| *Euphysa brevia* (Uchida, 1947) | *Sarsia brevia* | four equal tentacles with four abaxial clusters of nematocysts, including terminal cluster | - | umbrella bell-shaped | without apical canal | Uchida, 1947 |
| *Euphysa flammea* (Hartlaub, 1902) |  | with four tentacles irregularly moniliform, all alike in adult, but developed in succession | - | umbrella bell-shaped | without apical canal | Schuchert, 2010 |
| *Euphysa intermedia* (Schuchert, 1996) | *Corymorpha intermedia* | a single moniliform tentacle with around ten annular and one terminal nematocyst clusters | absent | with apical process of variable shape and height | without apical canal | Schuchert, 1996 |
| *Euphysa japonica* (Maas, 1909) | *Sarsia japonica* | four equal tentacles, nematocysts are scattered in the proximal part, the distal two-thirds with annular clusters of nematocysts | four equal tentacles | bell of strikingly high shape with flat upper part | without apical canal | Maas, 1909 |
| *Euphysa peregrina* (Murbach, 1899) | *Hypolytus peregrinus* | - | - | - | - | Murbach, 1899 |
| *Euphysa problematica* Schuchert, 1996 |  | four identical short tentacles with about 10 nematocyst clusters and a large terminal cluster | four identical short tentacles | umbrella almost spherical | a small apical chamber | Schuchert, 1996 |
| *Euphysa ruthae* Norenburg & Morse, 1983 |  | - | - | - | - | Norenburg and Morse, 1983 |
| *Euphysa scintillans* Gershwin, Zeidler & Davie, 2010 |  | main tentacle with  about 20–30 abaxial nematocyst clusters | three tentacles reduced  to mere rudimentary bulbs, with pigmented  core | with a small, dome-shaped  bell | without apical canal | Gershwin et al., 2010 |
| *Euphysa tentaculata* Linko, 1905 |  | usually with three tentacles, irregularly moniliform. One tentacle longer and thicker than others | two shorter and thinner tentacles irregularly moniliform on bulbs closest to bulb with main tentacle | quite globular with rounded apex | without apical canal | Schuchert, 2010 |
| *Euphysa tetrabrachia* Bigelow, 1904 |  | single long tentacle, moniliform with about 6-8 rings of nematocysts | the other  Three are equally developed, moniliform with about three rings of nematocysts on each | pear-shaped, with a low and broad apical  projection | without apical canal | Bigelow, 1904 |
| *Euphysa vervoorti* Brinckmann-Voss & Arai, 1998 |  | longest moniliform tentacle | three smaller moniliform tentacles | high, dome shaped exumbrella | without apical canal | Brinckmann-Voss & Arai, 1998 |
| *Margelopsis haeckelii* Hartlaub, 1897 |  | radial clusters of 4 to 7 (3-9) per bulb.  With nematocysts concentrated in rings and a small terminal knob, thus nearly moniliform | radial clusters of 4 to 7 (3-9) per bulb. With nematocysts concentrated in rings and a small terminal knob, thus nearly moniliform | with rounded apex, without apical projection | apical chamber (?): the endoderm base of the  manubrium extends through the apical gelatinous layer to the apex (in Werner, 1955: fig. 1); with apical canal according to diagnose (Schuchert, 2006), with  apical process (our data) | Werner, 1955; Schuchert, 2006;  Our data |

References:

Agassiz L. 1862. Contributions to the natural history of the United States of America. Little Brown, Boston. 4:1–380. *available online at* https://www.biodiversitylibrary.org/page/16068829

Alder J. 1857. VII. A Catalogue of the Zoophytes of Northumberland and Durham. By Joshua Alder, Esq. Read, at the Anniversary Meeting of the Club, May 15, 1856. From the “Transactions of the Tunesyde naturalists’ field club”. Newcastle-upon-Tyne: F.&W. Dodsworth, Collingwood street. 1–72 (93–154). *available online at*  https://archive.org/details/b30473858/mode/1up

Allman GJ. 1876. Diagnoses of new genera and species of Hydroida. *Journal of the Linnean Society of London* 12:251–284. *available online at* https://www.biodiversitylibrary.org/page/31981814

Bigelow HB. 1904. Medusae from the Maldive Islands. *Bulletin of the Museum of Comparative Zoology at Harvard College* 39:245–269. *available online at* https://www.biodiversitylibrary.org/page/4199303

Bouillon J. 1978. Hydromeduses de la mer de Bismarck (Papouasie, Nouvelle-Guinée). Partie 1: Anthomedusae Capitata (Hydrozoa - Cnidaria). *Cahiers de Biologie Marine* 19:249–297.

Brinckmann-Voss A, and Arai MN. 1998. Further notes on Leptolida (Hydrozoa: Cnidaria) from Canadian Pacific waters. *Zoologische Verhandelingen* 323(5):37–68.

Brinckmann-Voss A, and Calder DR. 2013. Zyzzyzus rubusidaeus (Cnidaria, Hydrozoa, Tubulariidae), a new species of anthoathecate hydroid from the coast of British Columbia, Canada. *Zootaxa* 3666(3):389–397.

Brooks WK. 1883. List of medusae found at Beaufort, N.C., during the summers of 1880 and 1881. *Studies from the Biological Laboratory, Johns Hopkins University* 2:135–146. *available online at* http://www.archive.org/stream/studiesfrombiol00martgoog#page/n152/mode/1up

Browne ET. 1916. Medusae from the Indian Ocean (collected by Prof. Stanley Gardiner in H.M.S. Sealark in 1905). In: The Percy Sladen Trust Expedition to the Indian Ocean. In: Transactions of the Linnean Society of London, Zoology 17: 169–209. *available online at* https://www.biodiversitylibrary.org/page/25256788

Clark SF. 1877. Report on the hydroids collected on the coast of Alaska and the Aleutian Islands by W.H. Dall, U.S. Coast Survey, and party, from 1871 to 1874 inclusive. *Proceedings of the Academy of Natural Sciences of Philadelphia* 28:209–235. *available online at* https://biodiversitylibrary.org/page/26298960

Du F-Y, Xu Z-Z, Huang J-Q, Guo D-H. 2012. Studies on the medusae (Cnidaria) from the Beibu Gulf in the northern South China Sea, with description of three new species. *Acta Zootaxonomica Sinica* 37:506–519. *available online at* http://eng.med.wanfangdata.com.cn/PaperDetail.aspx?qkid=dwfl&qcode=dwfl201203006

Du F-Y, Lin Z-J, Xu Z-Z, Huang J-Q, Guo D-H. 2013. Three new species of hydroidomedusae (Cnidaria) from the Meiji Reef and Daya bay, South China Sea. Acta Zootaxonomica Sinica 38(4):749–755.

Fraser CM. 1941. New species of hydroids, mostly from the Atlantic Ocean, in the United States National Museum. Proceedings of the United States National Museum 91:77–89. *available online at* http://www.biodiversitylibrary.org/item/32533#103

Genzano G, Rodriguez C, Pastorino G, Mianzan H. 2009. The hydroid and medusa of *Corymorpha januarii* (Cnidaria: Hydrozoa) in temperate waters of the Southwestern Atlantic Ocean. *Bulletin of Marine Science* 84(2):229–235.

Gershwin L-A, Zeidler W, Davie PJF. 2010. Medusae (Cnidaria) of Moreton Bay, Queensland, Australia. Memoirs of the Queensland Museum 54: 47–108.

Hamond R. 1974. Some medusae and other Hydrozoa from the Indian Ocean and the Bass Strait. *J. nat. Hist*. 8:549–561.

Hargitt CW. 1924. Hydroids of the Philippine Islands. *Philippine Journal of Science* 24:467–507.

Hickson SJ, and Gravely FH. 1907. II Hydroid Zoophytes. National Antarctic Expedition 3: 1–33. *available online at* https://www.biodiversitylibrary.org/page/854217

Hirohito Emperor of Japan. 1988. The hydroids of Sagami Bay. (Part 1. Athecata). *Publs Biol. Lab., Imp. Household, Tokyo* 1–179.

Huang J. 1999. Three new species of genus *Euphysora* from China seas (Hydrozoa: Anthomedusae, Corymorphidae). *Acta Oceanol. Sinica* (English Edit.) 18(3):435–441.

Huang J-Q, Xu Z-Z, Lin M, Guo D-H, Wang Ch-G, Xue W-L. 2012. One new genus, two new species and one new record of Corymorphidae from the South China Sea (Anthomedusae, Capitata). *Acta Zootaxonomica Sinica* 37:520–524.

Ikeda J. 1909. On a new species of *Corymorpha* from Japan. Annotnes zool. japon. 7(3):153–164.

Kavvamura M, and Kubota S. 2005. First occurrence of *Euphysora gemmifera* (Cnidaria, Hydrozoa, Corymorphidae) in Japan. *Biogeography* 7:31–33.

Kramp PL. 1928. Papers from Dr. Mortensen's Pacific Expeditions 1914-1916, XLIII. Hydromedusae 1. Anthomedusae. *Vidensk. Meddr dansk naturh. Foren.* 85:27–64.

Kramp PL. 1948. Trachymedusae and Narcomedusae from the "Michael Sars" North Atlantic deep-sea Expedition 1910, with additions on Anthomedusae, Leptomedusae, and Scyphomedusae. Report on the scientific results of the "Michael Sars" north Atlantic deep-sea expedition 1910. 5(9):1–23.

Kramp PL. 1957. Hydromedusae from the Discovery collections. *Discovery Rep.* 29:1–128. *available online at* http://www.biodiversitylibrary.org/page/5568717#page/15/mode/1up

Kramp PL. 1959. The Hydromedusae of the Atlantic Ocean and adjacent waters. *Dana Report* 46:1–283.

Kramp PL. 1961. Synopsis of the medusae of the world. *Journal of the marine biological Association of the United Kingdom* 40:7–382.

Kramp PL. 1962. Medusae of Vietnam. *Videnskabelige Meddelelser fra Dansk naturhistorisk Forening i København* 124:305–366.

Kramp PL. 1965. The hydromedusae of the Pacific and Indian Oceans. *Dana Report* 68:1–162.

Kramp PL. 1968. The hydromedusae of the Pacific and Indian Oceans. *Dana Report* 72:1–200.

Lin M., Xu Z.-Z., Huang J.-Q., Nurul F., Guo D.-H., Wang C.-G., Chen B. (2013). Two new species of Anthomedusae from the Bitung Strait, Indonesia (Cnidaria). Acta Zootaxonomica Sinica. 38(2): 246–250.

Liu ZY, Yang YY, Xu ZZ, Huang JQ. 2022. A new species of medusae from Luoyuan Bay, Fujian, China. Zoological Systematics 47(4):345–348.

López-Pérez A, Reyes-Bonilla H, Calderón-Aguilera LE, Cruz-Piñón G, Cupul-Magaña A, Medina-Rosas P, Mora-Vallín Z, Meléndez-Rosas R. 2022. Cnidarios (Cnidaria). In Bastida-Zavala JR, & del Socorro García-Madrigal M (Eds.) “Invertebrados marinos y costeros del Pacífico sur de México”, Universidad del Mar Puerto Ángel, Oaxaca, pp. 31–53.

Maas O. 1905. Die Craspedoten Medusen der Siboga-Expeditie. Siboga Expeditie 10:1–84. *available online at* https://www.biodiversitylibrary.org/page/11712344

Maas O. 1909. Japanische medusen. Series: Beiträge zur naturgeschichte Ostasiens. Abhandlungen der bayerischen Akademie der Wissenschaften, mathematisch physikalische Classe, Supplement Band 1:1–53. available online at https://www.biodiversitylibrary.org/page/5542853

Mayer AG. 1894. An account of some medusae obtained in the Bahamas. In: Cruise of the Steam Yacht "Wild-Duck" in the Bahamas, January to April 1893, in charge of Alexander Agassiz. Bulletin of the Museum of comparative Zoölogy of Harvard College 25(11):235–242. *available online at* http://www.biodiversitylibrary.org/item/24909

Mayer AG. 1910. Medusae of the World. *Published by the Carnegie Institution of Washington*. Publication No 109, Vol. 1, *available online at* https://doi.org/10.5962/bhl.title.5996

Murbach L. 1899. Hydroids from Wood's Hole. *Hypolytus peregrinus*, a new unattached marine hydroid: *Corinitis Aga*ssizii and its medusa. *Q. Jl microsc. Sci*., n. ser. 42. 3:341–360.

Nawrocki AM, Collins AG, Hirano YM, Schuchert P, Cartwright P. 2013. Phylogenetic placement of *Hydra* and relationships within Aplanulata (Cnidaria: Hydrozoa). *Molecular Phylogenetics and Evolution* 67(1):60–71.

Norenburg, J.L. and Morse, M.P., 1983. Systematic implications of Euphysa ruthae n. sp.(Athecata: Corymorphidae), a psammophilic solitary hydroid with unusual morphogenesis. Transactions of the American Microscopical Society, pp.1-17.

Ruthensteiner B, Reinicke GB, & Straube N. 2008. The type material of Hydrozoa described by Eberhard Stechow in the Zoologische Staatssammlung München. *Spixiana* 31(1):3–27.

Sars M. 1835. Beskrivelser og Iagttagelser over nogle moerkelige eller nye i Havet ved den Bergenske Kyst levende Dyr af Polypernes, Acalephernes, Radiaternes, Annelidernes og Molluskernes classer, med en kort Oversigt over de hidtil af Forfatteren sammesteds fundne Arter og deres Forekommen. [book]. Thorstein Hallagers Forlag hos Chr. Dahl, R.S., xii + 81 pp., 15 plates, Bergen. *available online at* https://doi.org/10.5962/bhl.title.13017

Sars M. 1860. Udtog af en Afhandling, som med de tilhörende talrige Afbildninger er bestemt for naeste Hefte af Fauna littoralis Norvegiae, om Ammeslaegten Corymorpha og dens Arter samt de af disse opammede Meduser. Forhandlinger i Videnskapsselskapet i Kristiania 1859. 96–105. *available online at* https://www.biodiversitylibrary.org/page/22192331

Sassaman C, and Rees JT. 1978. The life cycle of *Corymorpha* (= *Euphysora*) *bigelowi* (Maas, 1905) and its significance in the systematics of corymorphid hydromedusae. *The Biological Bulletin* 154(3):485–496.

Schuchert P. 1996. The marine fauna of New Zealand: Athecate hydroids and their medusae (Cnidaria: Hydrozoa). *New Zealand oceanographic institute memoir* 106:1–159.

Schuchert P. 2006. The European athecate hydroids and their medusae (Hydrozoa, Cnidaria): Capitata part 1. *Revue suisse de Zoologie* 113(2):325–410. DOI: 10.5962/bhl.part.80356

Schuchert P. 2010. The European athecate hydroids and their medusae (Hydrozoa, Cnidaria): Capitata part 2. *Revue suisse de Zoologie* 117(3):337–555.

Schuchert P, Collins R. 2021. Hydromedusae observed during night dives in the Gulf Stream. Revue suisse de Zoologie 128(2):237–356. *available online at* https://doi.org/10.35929/rsz.0049

Silveira FD, Migotto AE. 1992. Rediscovery of *Corymorpha januarii* Steenstrup, 1854 (Hydrozoa, Corymorphidae) on the southeastern and southern coasts of Brazil. *Steenstrupia* 18(4):81–9.

Stechow E. 1932. Neue Hydroiden aus dem Mittelmeer und dem Pazifischen Ozean, nebst Bemerkungen über einige wenig bekannte Formen. *Zoologischer Anzeiger* 100:81–92.

Steenstrup JJS. 1855. En ny og tropisk Art af Smaagoplernes Ammeslaegt: *Corymorpha* Sars (*Corym. Januarii* Stp.). Vidensk. Meddr dansk naturh. Foren. 1–3:46–48.

Svoboda A, and Stepanjants SD. 2001. Redescription of two Antarctic Corymorphidae species and the reestablishment of the genus *Monocaulus* (Cnidaria: Hydrozoa). *Marine Ecology* 22(1‐2):53–70.

Torrey HB. 1902. The Hydroida of the Pacific Coast of North America. University of California Publications Zoology. 1: 1–104. *available online at* https://biodiversitylibrary.org/page/29942277
Uchida T. 1927. Studies on Japanese hydromedusae. I. Anthomedusae. Journal of the Faculty of Science, Imperial University of Tokyo, Section IV, Zoology. 1:145–241.

Uchida T. 1947. Some medusae from the Central Pacific. *Journal of the Faculty of Science, Imperial University of Tokyo, Zoology* 7:297–319.

Vanhöffen E. 1911. Die Anthomedusen und Leptomedusen der Deutschen Tiefsee Expedition 1898-1899. *Wissenschaftliche Ergebnisse der deutschen Tiefsee Expedition Valdivia* 19:193–233.

Vargas-Hernández JM, and E. Ochoa-Figueroa. 1991. Un nuevo género y descripción de una nueva especie para la familia Tubulariidae (Hidrozoa: Anthomedusae) en el Pacífico méxicano. A new genus and description of a new species for the family Tubulariidae (Hydrozoa, Anthomedusae) in the Mexican Pacific. *Brenesia* 33:75–80.

Vervoort W. 2009. *Corymorpha tomoensis* Ikeda, 1910 (Cnidaria, Hydrozoa): first record of a corymorphid hydropolyp from Indonesian waters and a review of the species of Corymorpha M. Sars, 1835. *Zoologische Mededelingen* 83(21):759–776.

Wang X, Lin K, Xu Z, Guo D, Huang J. 2019. Some new Hydroidomedusa (Cnidaria) from the northern South China Sea. *Zoological Systematics* 44(3):191–205. DOI: 10.11865/zs.201923

Watson JE. 2008. *Corymorpha rubicincta*, a new Hydroid (Hydrozoa, Anthoathecata, Corymorphidae) from Port Phillip, Australia. *Memoirs of Museum Victoria* 65:185–188.

Werner B. 1955. On the development and reproduction of the anthomedusan *Margelopsis haeckeli* Hartlaub. *Annals of the New York Academy of Sciences* 62(1):3–29. doi: 10.1111/j.1749-6632.1955.tb35352.x

Xu Z, Huang J. 2003. On new species and records of *Euphysora* in Taiwan Strait and its adjacent waters J. *Oceanogr. Taiwan Strait = Taiwan Haixia* 22(2):136–144.

Xu ZZ, and Huang JQ. 2006. On new genus, species and record of Laingiomedusae and Anthomedusae in Fujian coast (Cnidaria, Hydroidomedusae). *Journal of Xiamen University Natural Science* 45:233–249.

Xu Z-Z,Huang J-Q, Lin M, Guo D-H, Wang C-G. 2014. The superclass Hydrozoa of the Phylum Cnidaria in China. China Ocean Press, Bejing. 1:1–456, 2:495–945.

Yamada M, Konno K, Kubota S. 1977. On a new athecate hydroid, *Fukaurahydra anthoformis* n. gen. n. sp., from northern Japan. *Proc. jap. Acad. Sci.* 53(3):151–154.
